# Virtual versus in-person sexual rehabilitation for prostate cancer survivors: a retrospective cohort study comparing the Prostate Cancer Rehabilitation Clinic (PCRC) and the True North Sexual Health and Rehabilitation eClinic (SHAReClinic)

**DOI:** 10.1093/sexmed/qfaf088

**Published:** 2025-11-06

**Authors:** Andrew G Matthew, Steven Guirguis, Taylor Incze, Dalia Peres, Richard J Wassersug, Lauren Walker, John W Robinson, Deborah McLeod, Stacy Elliott, Antionio Finelli, Neil Fleshner, Leah Jamnicky, Shiyi Chen, Dean Elterman

**Affiliations:** Department of Surgical Oncology, Princess Margaret Cancer Centre, University Health Network, Toronto, ON M5G 1Z5, Canada; Department of Surgical Oncology, Princess Margaret Cancer Centre, University Health Network, Toronto, ON M5G 1Z5, Canada; Department of Surgical Oncology, Princess Margaret Cancer Centre, University Health Network, Toronto, ON M5G 1Z5, Canada; Department of Surgical Oncology, Princess Margaret Cancer Centre, University Health Network, Toronto, ON M5G 1Z5, Canada; Cellular and Physiological Sciences, University of British Columbia, Vancouver, BC V5Z 1M9, Canada; Department of Oncology, University of Calgary, Calgary, AB T2N 1N4, Canada; Department of Oncology, University of Calgary, Calgary, AB T2N 1N4, Canada; School of Nursing, Dalhousie University, Halifax, NS B3H 2Y9, Canada; BC Center for Sexual Medicine, Vancouver Coastal Health Authority, Vancouver, BC V6Z 2K5, Canada; Department of Surgical Oncology, Princess Margaret Cancer Centre, University Health Network, Toronto, ON M5G 1Z5, Canada; Department of Surgical Oncology, Princess Margaret Cancer Centre, University Health Network, Toronto, ON M5G 1Z5, Canada; Department of Surgical Oncology, Princess Margaret Cancer Centre, University Health Network, Toronto, ON M5G 1Z5, Canada; Department of Biostatistics, Princess Margaret Cancer Centre, University Health Network, Toronto, ON M5G 2M9, Canada; Division of Urology, University Health Network, Department of Surgery, University of Toronto, Toronto, ON M5T 2SB, Canada

**Keywords:** sexual dysfunction, oncosexology, sexual health, prostate cancer, virtual care, survivorship

## Abstract

**Background:**

Sexual dysfunction is a common and distressing consequence of prostate cancer (PCa) treatment, yet few healthcare institutions offer comprehensive, systematic care, limiting equitable access. Virtual models may improve accessibility and efficiency without sacrificing effectiveness.

**Aim:**

To assess whether outcomes in the virtual Sexual Health and Rehabilitation eClinic (SHAReClinic) were comparable to those in the in-person Prostate Cancer Rehabilitation Clinic (PCRC) in improving sexual health outcomes for PCa survivors.

**Methods:**

A retrospective cohort chart review was conducted on PCa patients enrolled in either the PCRC or SHAReClinic between September 2017 and August 2018, with data collected 12 months post-treatment. Clinic assignment was based on standard care pathways. Sexual health outcomes were measured using the Sexual Health Inventory for Men (SHIM), Sexual Distress Scale (SDS), Male Sexual Health Questionnaire (MSIS), and Expanded Prostate Cancer Index Composite (EPIC-26). Pro-erectile medication usage was also analyzed.

**Outcomes:**

Primary outcomes were SHIM and SDS scores. Secondary outcomes included MSIS, EPIC-26 scores, and pro-erectile medication use as an indicator for adherence and ongoing sexual activity.

**Results:**

Among 98 PCa patients (55 PCRC, 43 SHAReClinic), no significant differences were found in SHIM and SDS scores. While partnered PCRC participants reported significantly higher intimacy on the MSIS compared to partnered SHAReClinic participants (*P* = .042), no significant differences were found on the EPIC-26 sexual health domain. Pro-erectile medication use was similar in both groups. Multivariable analyses showed comparable outcomes in sexual function, distress, and health-related quality of life, except for intimacy.

**Clinical Implications:**

SHAReClinic yields comparable outcomes to PCRC and provides an effective, resource-efficient alternative to in-person sexual rehabilitation for PCa patients, particularly in settings with limited accessibility or resources.

**Strengths and Limitations:**

This study provides a comprehensive assessment of sexual health outcomes; however, the small sample size limits generalizability. In addition, there was a significant imbalance in treatment modality, with radiation patients represented only in the SHAReClinic cohort. Further research in larger, more diverse populations with longer follow-up is needed to confirm these findings and better understand intimacy differences.

**Conclusion:**

SHAReClinic demonstrates outcomes comparable to PCRC in managing sexual dysfunction in PCa patients, offering a viable and accessible option for sexual rehabilitation.

## Introduction

Prostate cancer (PCa) is the most commonly diagnosed non-skin cancer and the third leading cause of cancer-related deaths among men in Canada.^1^ Each year, over 25 000 men are diagnosed with PCa, accounting for 22% of all new cancer cases in Canada.^2^ Advances in early detection and treatment have improved PCa prognosis, with a 5-year relative survival rate now reaching 91%.^2^ However, treatment-related sexual dysfunction (SD) remains common, often necessitating ongoing post-treatment care.^3–10^

Post-treatment SD may include erectile dysfunction (ED), reduced libido, ejaculatory dysfunction, penile shortening or shrinkage, and urinary incontinence. Erectile dysfunction rates can be as high as 78%-87% in men 2-15 years after radical prostatectomy (RP).^11^ Research has shown that up to 60% of men who had normal erectile function prior to RP (ie, firm enough for penetration) report ED 2 years following treatment.^12^ Recovery timelines vary, and, despite gradual improvements, more than three-quarters of patients continue to experience SD-related distress 2 years post-RP.^13^

Beyond ED, the psychological and relational impact of SD-related issues can significantly impact emotional well-being and overall health-related quality of life (HRQoL).^7^^,^^8^^,^^14–16^ Studies indicate that 30%-50% of PCa survivors report unmet needs in areas like sexuality, psychological support, healthcare services, and information.^17^ SD can contribute to long-term difficulties in relationships, with partners often experiencing increased stress, decreased emotional intimacy, and overall marital quality declines.^18–20^ Compared to healthy, age-matched controls, men post RP report lower sexual confidence, reduced intimacy, greater anxiety, and a diminished sense of masculinity.^21^ Some men noted that declines in sexual function were strongly associated with treatment regret, emphasizing how central sexual health is to quality of life and decision satisfaction.^22^

Given the physical, psychological, and relational challenges posed by PCa treatments, healthcare providers have developed programs to address SD.^18^^,^^23^ Historically, interventions focused mainly on the physiological aspects of SD, using medical treatments such as phosphodiesterase type 5 inhibitors (PDE5is). However, experts argue that medical treatments alone are insufficient for comprehensive rehabilitation and long-term satisfaction.^18^ Instead, biopsychosocial interventions that combine medical, psychosocial, and sexual counseling are recommended to enhance sexual well-being.^17^^,^^24^^,^^25^ Evidence suggests that this holistic approach leads to improvements, such as reduced stress, greater use of pro-erectile aids, more realistic recovery expectations, and higher relationship satisfaction.^17^^,^^20^^,^^25–27^

To meet the demand for sexual rehabilitation, the Princess Margaret Cancer Centre (PM) launched the Prostate Cancer Rehabilitation Clinic (PCRC) in 2009. This in-person clinic offered multi-disciplinary sexual health care and counseling involving urologists, nurses, psychologists, and sexual health professionals. It was integrated into standard care to ensure continuity during the transition from pre- to post-treatment. In 2017, the True North Sexual Health and Rehabilitation eClinic (SHAReClinic) was introduced to improve accessibility for patients who faced barriers to attending the PCRC, such as geographic distance, time constraints, or stigma. SHAReClinic offers a virtual version of the biopsychosocial care provided by the PCRC and includes tailored educational modules, a health library, tracking and feedback systems, and access to a sexual health coach through asynchronous chat-based communication. The PCRC closed in 2020 due to the COVID-19 pandemic, and SHAReClinic subsequently replaced the PCRC as usual care at PM.

The primary objective of this study is to retrospectively evaluate the sexual health of patients attending either PCRC or SHAReClinic 1 year after enrollment and to compare outcomes between the 2 clinics. Secondary objectives include evaluating erectile function, relationship and intimacy levels, and adherence to pro-erectile medications for patients participating in either program post-PCa treatment.

## Methods

### Design

A retrospective cohort chart review assessing clinical data and Patient-Reported Outcome Measures (PROMs) collected during visits to the PCRC and SHAReClinic was conducted. Institutional ethics review board approval was obtained (Study ID: 21-6220). The review included patients treated for localized PCa at PM in 2017-2018 who attended 1 of the 2 clinics. Data were collected at baseline (pre-treatment), and at 6 weeks, 6 months, and 12 months post-treatment. This study aimed to compare outcomes at 12 months using a retrospective cohort design.

### Participants

#### Inclusion/exclusion criteria

Eligible participants included partnered and unpartnered individuals scheduled for first-line treatments of localized PCa, either through RP (open or robotic) or radiation (brachytherapy, external beam), who were hormone and/or chemotherapy-naïve, aged 18 years or older, and attended either the PCRC or SHAReClinic during September 2017 to August 2018 for 12 months.

Exclusion criteria included lack of English proficiency, nitrate therapy, contraindications to PDE5is, prior PCa treatment, medical conditions in the patient or partner that precluded safe sexual activity, or incomplete or missing clinical documentation.

### Participant selection

The study coordinator screened clinic lists from participating physicians’ (uro-oncologists and radiation oncologists) to identify eligible participants. Screening specifically targeted patients returning for prostate biopsy results or those with a confirmed PCa diagnosis. During their regular clinical appointments, the treating physician approached eligible patients (and if present, their partners). Patients opting for RP surgery or radiation were provided an introduction letter, which offered them a choice between standard in-person care at the PCRC or joining the newly established web-based sexual health and rehabilitation intervention, SHAReClinic. Those who declined SHAReClinic were referred to PCRC as standard care.

The SHAReClinic was launched as a real-world implementation assessment to provide timely care. This approach eliminated a structured recruitment phase, instead relying on a real-world self-selection process where patients opted into SHAReClinic during routine clinical care.

Patients interested in SHAReClinic were referred to the study coordinator, who shared the study introduction letter and informed consent for the SHAReClinic feasibility study.^26^ Both documents were also provided to their partners.

Consenting patients, along with partners, received instructions on how to access SHAReClinic (https://sharec.truenth.ca/) and were asked to register using their email and a secure password. Single men registered as an individual, while partnered men registered as a couple.

### Interventions

#### Prostate Cancer Rehabilitation Clinic

The PCRC was a specialized clinic, fully covered under the Canadian public healthcare system, that provided comprehensive support and treatment for men who had undergone RP for PCa^18^. It was established in 2009 at the PM in Toronto, Ontario, with the aim of improving sexual functioning and maintaining intimacy after PCa treatment through a biopsychosocial approach. This involved 2 main components: a biomedical component (erectile rehabilitation), focusing on the long-term penile health or short-term erectile function as per patient preference, via pro-erectile therapies; and a psychosocial component (intimacy maintenance), aimed at supporting the emotional connection and relationship closeness between couples. The psychosocial intervention targeted enhancing relationship satisfaction, reducing sexual distress, increasing sexual confidence, and supporting both penetrative and non-penetrative sexual activities.

The clinic was staffed by a multidisciplinary intervention team including urologists, sexual health coaches, psychologists, nurses, and researchers, who collaborated to provide holistic sexual health care. Patients and their partners were introduced to the PCRC preoperatively and were provided with information about the services offered. Interested patients were scheduled for a clinic appointment 6-8 weeks after their PCa treatment.

The assessment process at the PCRC involved a structured clinical interview to determine the patient’s current and past sexual functioning, as well as their specific concerns and goals. PROMs were also collected at each appointment to assess the effectiveness of the interventions.

The PCRC followed a scheduled series of 7 clinic visits across 2 years, including 1 pre-treatment visit and 6 follow-ups. The program collected data for program evaluation to examine the clinic’s effectiveness and outcomes. A full description of the PCRC can be found at Matthew et al.^18^

#### SHAReClinic

SHAReClinic is an innovative online biopsychosocial clinic designed to provide sexual health and rehabilitation support for men treated for localized PCa^28^. Like PCRC, SHAReClinic is publicly funded and incurs no cost to patients. Building on the biopsychosocial approach pioneered at the now-retired PCRC, SHAReClinic integrates both biomedical and psychosocial elements of sexual recovery. The development of this virtual clinic was driven and supported by the Movember Foundation, whose mission includes promoting equitable access to care by overcoming geographic and logistical barriers.

By providing an accessible and convenient online platform, SHAReClinic addresses challenges associated with in-person clinics and delivers comprehensive sexual health and rehabilitation support. Unlike traditional telehealth programs that rely heavily on synchronous interactions, SHAReClinic primarily uses asynchronous care delivery, including self-guided web-based modules, a digital health library, and secure asynchronous messaging with a sexual health coach and limited synchronous communication if needed. The modules are tailored based on patients’ treatment type, relationship status, and sexual orientation, making the clinic suitable for all people with prostate cancer.

The SHAReClinic intervention consists of 6 self-guided web-based clinic e-visits: 1 pre-treatment and 5 follow-up visits over a 1-year period post–PCa treatment. The visit schedule reflects critical psychosocial and biomedical sexual health milestones in PCa recovery.^28^ The e-visits offer information and guidance on various topics related to sexual health, rehabilitation, and intimacy maintenance. Patients also have access to a digital health library featuring additional resources. Patients have continued access to the health library and their sexual health in the second year and continued access as needed beyond the second year.

Upon registration, patients are paired with a sexual health coach who plays a crucial role in the intervention. These coaches are healthcare professionals, including social workers, nurses, psychology trainees, and radiation therapists, who have completed the True North Sexual Health and Rehabilitation eTraining (SHAReTraining) course—a specialized 20-hour online program designed to build knowledge and skills in managing sexual health concerns for PCa patients and their partners. A detailed description of the SHAReTraining course is available in Matthew et al.^29^

Sexual health coaches introduce patients to the platform, answer questions, and provide tailored support throughout their recovery process. They exchange messages with participants to address their concerns and provide personalized guidance but do not deliver clinical diagnoses or medical treatment. It is estimated that the sexual health coach will spend approximately 4–6 hours per patient for the 2-year program, highlighting both the cost-effectiveness of the model and the reduction in provider workload.^28^

Core topics covered in the e-clinic visit include: education and normalizing sexual health rehabilitation, post-treatment expectations, intimacy and passion, challenges to naturalness and spontaneity, adaptation to sexual response changes, performance anxiety, masculinity, partner concerns, communication, adaptation to long-term use of pro-erectile therapy, and exploration of non-intercourse sexual activity.

A feasibility study conducted on the SHAReClinic demonstrated its acceptability and promise as a viable, resource-efficient approach to improving the sexual well-being of PCa patients.^28^ Participants showed high adherence, engagement, and satisfaction. A full description of the SHAReClinic can be found in Matthew et al.^28^

## Evaluation and measures

### Patient-reported outcomes

The SHIM and SDS were used as the primary measures for sexual health in PCa patients. Secondary measures included patient relationship and intimacy levels (MSIS), HRQoL (EPIC-26 Sexual Function Domain), erectile functioning, and participant adherence to pro-erectile medication use (see Table 1. Assessment Tools).

**Table 1:** Assessment Tools.

**Questionnaire**	**Purpose**	**Assessment role**
Demographics	Collect participant background information for contextual analysis and group comparison	Context and Group Equivalence
SHIM (Sexual Health Inventory for Men)	Evaluate erectile function and identify severity of dysfunction	Primary
SDS (Sexual Distress Scale)	Measure psychological distress related to sexual problems and dysfunction	Primary
MSIS (Miller Social Intimacy Scale)	Assess emotional closeness, trust, and communication within partnered relationships	Secondary
EPIC-26 (Expanded Prostate Cancer Index Composite-26) Sexual Function Domain	Assess the impact of prostate cancer and its treatment on sexual function, sexual bother, and HRQoL	Secondary
Medication Usage	Monitor pro-erectile therapy usage, frequency, and adherence	Secondary

### Assessment of primary outcomes

#### Sexual Health Inventory for Men

The SHIM, also known as the International Index of Erectile Function (IIEF-5), was developed and validated by Rosen and colleagues as a tool to assess erectile function and dysfunction in men.^30^^,^^31^ It consists of 5 self-report questions that specifically address various aspects of erectile function, such as the ability to achieve and maintain an erection, erection quality, and overall satisfaction with sexual activity. The SHIM is commonly used to assess the impact of PCa and its treatments on patients’ erectile function.

#### Sexual Distress Scale

The male SDS is a validated self-report questionnaire designed to assess an individual’s sexual distress related to sexual function and problems.^32^^,^^33^ While initially developed for females,^34^ it has been updated and validated for use in men and more specifically in men with PCa,^33^ making it a widely used clinical and research tool to evaluate sexual distress in this population. The SDS consists of 12 items that assess the level of distress or bother experienced by individuals across various aspects of their sexual function and sexual life.

### Assessment of secondary outcomes

#### Miller Social Intimacy Scale

The MSIS is a 17-item scale that assesses an individual’s perception of emotional closeness, trust, communication, and overall satisfaction with social intimacy in relationships.^35^ In this study, the full MSIS was administered only to participants who reported being in a partnered relationship at baseline, as the measure specifically captures experiences relevant to intimate partnerships.

#### Expanded Prostate Cancer Index Composite-26

The clinics used the EPIC-26 to assess the HRQoL of patients. The EPIC-26 is a well-known and validated questionnaire specifically designed for PCa patients. It evaluates the impact of PCa and its treatments on 5 domains: urinary function, bowel function, sexual function, and hormonal function, as well as related bother and overall quality of life.^36^^,^^37^

While the EPIC-26 is typically used to monitor changes over time and evaluate the specific effects of PCa and its treatments, this study focused solely on the sexual function domain of the questionnaire. This was done as both interventions exclusively addressed sexual function and did not directly address the other domains.

### Pro-erectile medication usage

A 2-item questionnaire was administered to assess patients’ use of pro-erectile therapies over the previous 4 weeks. This included both prescribed oral (eg, PDE5i) and injectable therapies (eg, intracavernosal injections). The questionnaire determined which therapies were utilized, the number of attempts made, and adherence during this period. This assessment allowed the clinical team to gauge the effectiveness of these treatments in improving erectile function and overall sexual well-being. Additionally, it helped identify patients’ needs and barriers to treatment adherence, facilitating personalized and targeted care.

### Data analysis

To help mitigate bias, we used validated patient-reported outcome measures and consistent eligibility criteria across groups. For each questionnaire, descriptive statistics were calculated where counts (proportions) were calculated for categorical variables and means (standard deviations) and medians (ranges) were estimated for continuous variables. To assess the differential impact of PCRC and SHAReClinic on the various questionnaire instruments (SHIM, SDS, MSIS, EPIC-26), univariable and multivariable linear regression models were utilized. Separate analyses were conducted for each outcome. Outliers for any variable at any time point were excluded from the analysis to prevent misrepresentation of central tendency and variance. Variables with *P* < .10 in the univariable analysis were included in the multivariable model. In addition, clinically critical variables, regardless of statistical significance, were included. Based on these criteria, ethnicity, relationship status, and job status were included in the final multivariable model. Analyses were conducted using available data; no imputation was performed for missing values. All statistical analyses were carried out using SAS 9.4 software (Cary, NC, USA), with statistical significance set at *P* < .05.

## Results

### Participant characteristics

A total of 98 patient charts had complete data, including both baseline and 12-month post-treatment reports: 55 from the PCRC group and 43 from the SHAReClinic group. No statistically significant differences were found in demographic variables between the 2 groups. Among the 98 patients, 68.67% identified as Caucasian, 90.63% were in a partnered relationship, 81.91% were married, and 65.22% were employed. The average age was 61.56 years. Treatment modality differed significantly between groups (*P* = .001). The PCRC cohort consisted entirely of RP patients, whereas the SHAReClinic cohort included both surgical (81%) and radiation (19%) patients. A detailed comparison between the 2 clinic groups is presented in Table 2.

**Table 2:** Summary of participants’ demographic information.

	**PCRC** (N = 55)	**SHAReClinic** (N = 43)	**Total** (N = 98)	***P* value**
**Age**				
Mean (SD)	60.47 (11.16)	62.98 (7.09)	61.56 (9.65)	.18
Median (range)	62.0 (42.0– 72.0)	64.5 (47.0– 74.0)	63.0 (42.0– 74.0)	.18
N (missing)	55 (0)	42 (1)	97 (1)	
**Ethnicity**				
Caucasian	25 (60.98%)	32 (76.19%)	57 (68.67%)	.14
Other	16 (39.02%)	10 (23.81%)	26 (31.33%)	
Missing	14	1	15	
**Marital status**				
Married	45 (88.24%)	32 (74.42%)	77 (81.91%)	.08
Other	6 (11.76%)	11 (25.58%)	17 (18.09%)	
Missing	4	0	4	
**Relationship status**				
In a relationship	50 (94.34%)	37 (86.05%)	87 (90.63%)	.29
Not in a relationship	3 (5.66%)	6 (13.95%)	9 (09.38%)	
Missing	2	0	2	
**Job**				
Not working	14 (28.57%)	18 (41.86%)	32 (34.78%)	.18
Working	35 (71.43%)	25 (58.14%)	60 (65.22%)	
Missing	6	0	6	
**Treatment type**				
Radical prostatectomy	55 (100.0%)	35 (81.39%)	90 (91.84%)	.001
Radiation therapy	0 (0.0%)	8 (18.60%)	8 (8.16%)	

### Questionnaires

#### Primary

##### Sexual Health Inventory for Men

**Figure 1:**
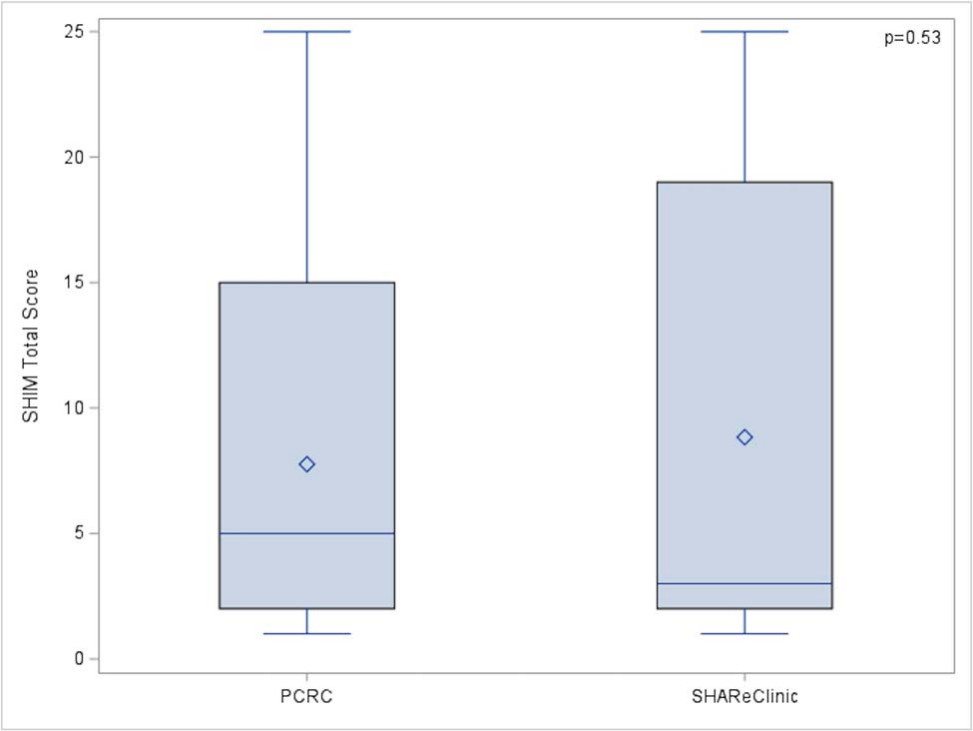
PCRC vs SHAReClinic SHIM Score.

The average SHIM total score did not differ between PCRC and SHAReClinic (7.76 ± 7.77 vs 8.84 ± 9.12, *P* = .53), see Figure 1. In the multivariable model, SHIM total score did not differ by clinic (β = 1.41, SE = 1.98, *P* = .48), after adjusting for other risk factors.

##### Sexual Distress Scale

**Figure 2:**
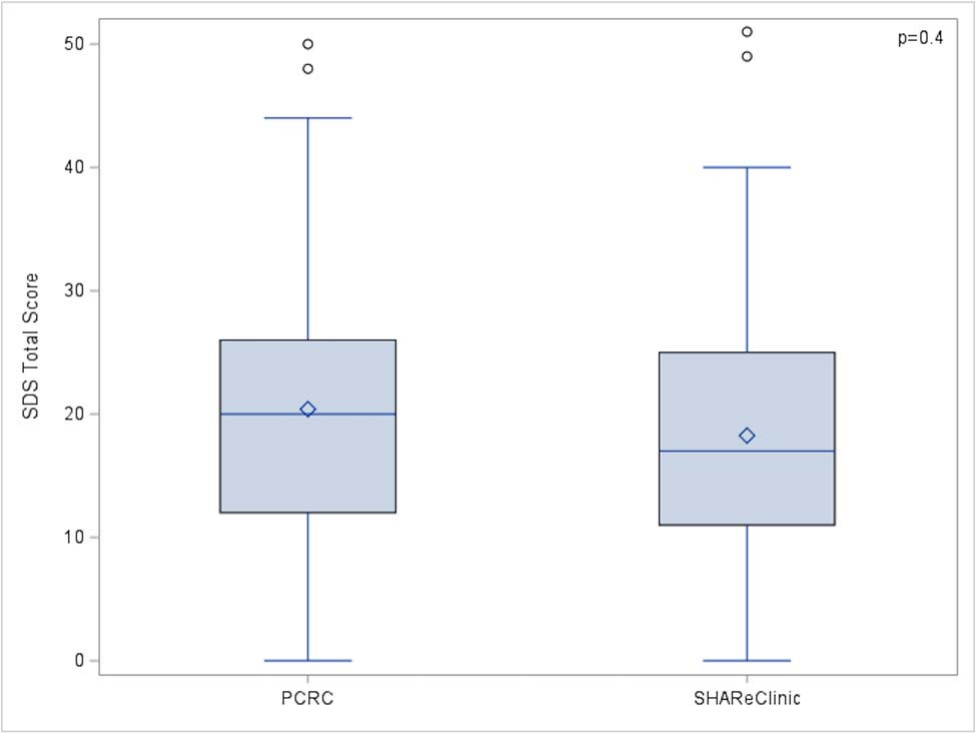
PCRC vs SHAReClinic SDS Score.

No statistically significant difference was found in terms of average SDS total score between PCRC and SHAReClinic (20.40 ± 12.69 vs 18.26 ± 12.01, *P* = .4) see Figure 2. In the multivariable model, SDS total score did not differ by clinic (β = 1.83, SE = 3.02, *P* = .55), after adjusting for other risk factors.

***Secondary***

#### Miller Social Intimacy Scale

**Figure 3:**
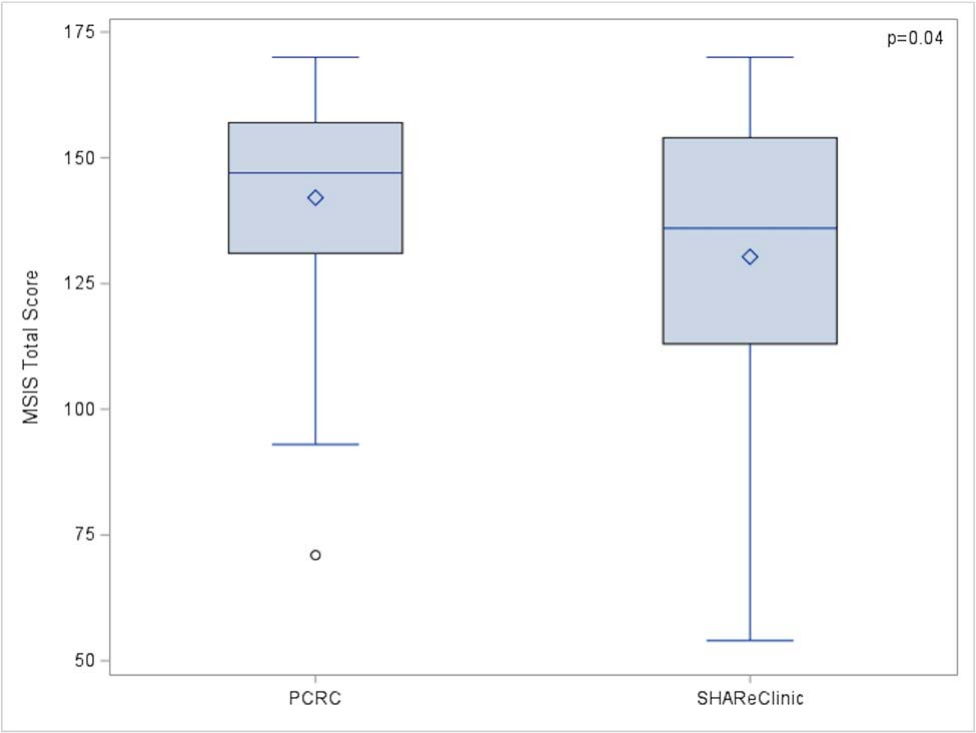
PCRC vs SHAReClinic MSIS Score.

The mean MSIS total score was significantly higher in the PCRC compared to the SHAReClinic (142.07 ± 21.13 vs 130.32 ± 29.02, *P* = .042), see Figure 3. In the multivariable model, patients in the PCRC showed a significantly higher MSIS total score than those in the SHAReClinic (β = 15, SE = 6.15, *P* = .02), after adjusting for other risk factors such as age, ethnicity, marital status, and job status. Caucasian patients showed a significantly higher MSIS total score than patients of other ethnicities (*P* = .01).

##### Expanded Prostate Cancer Index Composite-26

**Figure 4:**
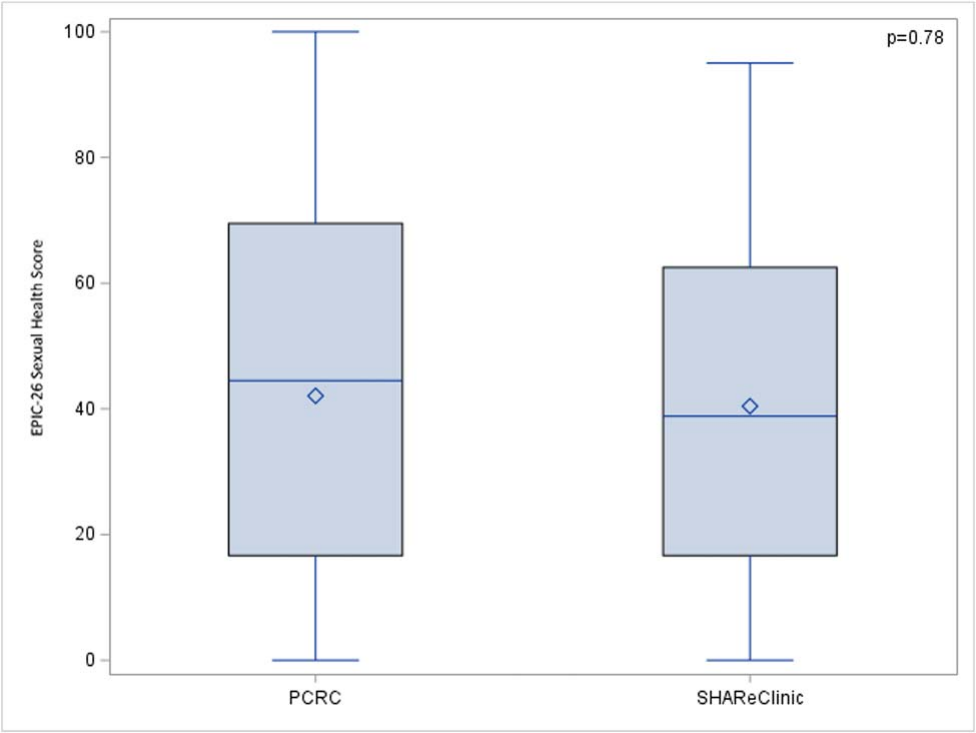
PCRC vs SHAReClinic EPIC-26 Sexual Health Score.

No statistically significant differences were found between PCRC and SHAReClinic in terms of Sexual Health Domain score (42.07 ± 29.30 vs 40.43 ± 28.39, *P* = .78), see Figure 4. Responses to item 9 of the EPIC-26 questionnaire (“How would you describe the usual QUALITY of your erections during the last 4 weeks?”) showed that SHAReClinic had similar treatment outcomes as PCRC (“sexually functional”: 11 [25.58%] vs 13 [25.49%]; “Firm enough for masturbation and foreplay”: 12 [27.91%] vs 13 [25.49%]; “Not at all, not firm enough for any sexual activity” 20 [46.51%] vs 25 [49.02%]). Responses to item 12 of the EPIC-26 questionnaire (“Overall, how big a problem has your sexual function or lack of sexual function been for you during the last 4 weeks?”) also suggested that SHAReClinic was comparable to the PCRC (“No problem, Very small problem, Small problem”: 26 [60.47%] vs 33 [66.00%]; “Moderate problem, Big problem”: 17 [39.53%] vs 17 [34.00%]). In the multivariable analysis, for EPIC-26 Sexual Health Domain score did not differ by clinic (β = -2.46, SE = 6.69, *P* = .71).

### Pro-erectile medication usage

**Figure 5:**
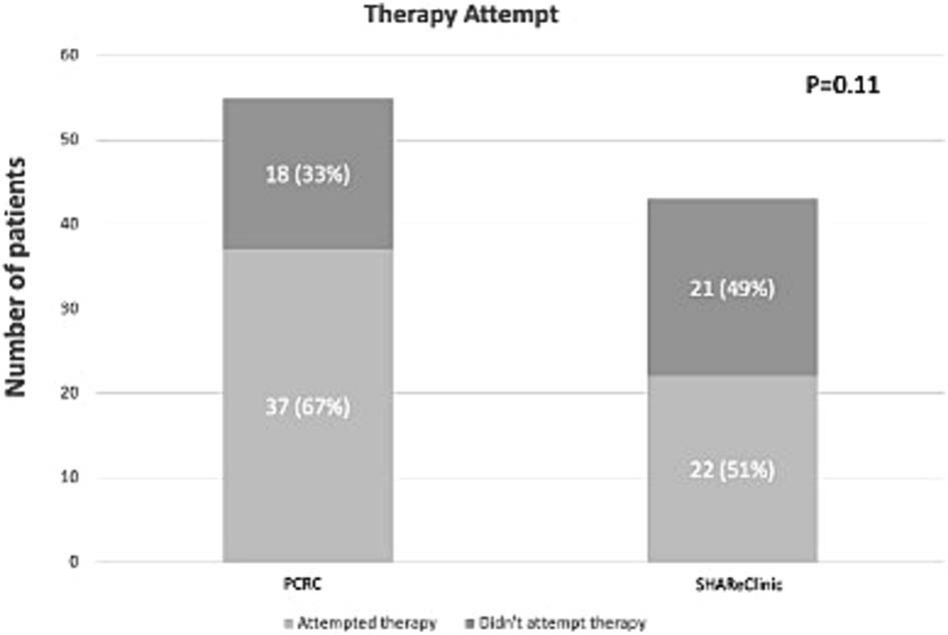
Pro-erectile medication therapy attempt.

The average number of pro-erectile medication therapy attempts did not significantly differ between PCRC and SHAReClinic (6.52 ± 7.38 vs 9.95 ± 6.91, *P* = .12), see Figure 5. Similarly, the proportion of patients attempting therapy did not differ between the clinics (37 [67.27%] vs 22 [51.16%], *P* = .11). Moreover, subsequent analysis through a multivariable logistic regression model, adjusting for other risk factors, also did not show a statistically significant difference in therapy use between clinics (odds ratio [95% CI] = 2.72 [0.99– 7.39], *P* = .05).

## Discussion

SD after PCa treatment is a common and significant issue for many patients, often affecting their overall quality of life. However, many cancer centers lack specialized sexual health clinics to address this concern. In response, the PCRC was established to provide comprehensive biopsychosocial sexual healthcare to PCa patients and their partners, following evidence-based guidelines and best practice models. To improve access for patients and couples who may face barriers like geographic distance, limited resources, or the financial costs of travel, the virtual SHAReClinic was developed. We conducted this study as a retrospective cohort comparison, to determine whether outcomes from the virtual were comparable to those from PCRC.

The study included 98 patients, 55 from the PCRC and 43 from the SHAReClinic. The majority of participants were Caucasian (68.67%), in a relationship (90.63%), married (81.91%), employed (65.22%), and had an average age of 61.56 years. These demographic characteristics were consistent across both groups, with no statistically significant differences between the 2 clinics.

The primary outcomes, erectile function (measured by the SHIM) and sexual distress (measured by the SDS), did not show significant differences between the PCRC and SHAReClinic. Most secondary outcomes also showed no significant variations. Analysis of the sexual function domain of the EPIC-26 revealed no notable differences between the 2 clinics, with both groups reporting similar improvements in erection quality, firmness, and overall sexual function. Pro-erectile medication usage patterns, including adherence and number of attempts, were also comparable between the 2 clinics, indicating similar effectiveness in supporting patients’ adherence to medication, a key aspect of post-treatment sexual rehabilitation. The continued use of pro-erectile medication over time also indicated that patients in both clinics were committed to using these aids, either for maintaining sexual intimacy post-treatment or to promote future sexual capacity. These finding suggest that both clinics provided comparable education and guidance regarding the use of pro-erectile aids. Contuined PDE5i use suggests sustained patient engagement, even when erectile function had not fully returned, highlighting the importance of ongoing support.

The sample included both partnered and unpartnered individuals; however, intimacy outcomes measured via the MSIS were analyzed only for partnered participants, as unpartnered participants did not complete the measure. A notable finding from the MSIS was that partnered patients in the PCRC reported significantly higher levels of intimacy in their relationships compared to those in the SHAReClinic. This difference was further supported by the multivariable model, which showed that Caucasian patients reported significantly higher MSIS scores than patients from other ethnic backgrounds. Further research should consider whether certain demographic groups are more likely to attend with their partners or whether cultural factors may influence perceptions of intimacy.

Despite these differences, both clinics showed similar outcomes in terms of sexual function, distress, and overall HRQoL. The results of this retrospective cohort study suggest that outcomes in the SHAReClinic were comparable to those in the in-person PCRC, supporting virtual care as an effective approach for managing SD in PCa survivors. Given that SHAReClinic is a virtual platform, these findings suggest it may provide a resource-efficient and accessible model of care without compromising patient satisfaction or clinical outcomes.

Our findings align with previous studies that have explored the effectiveness of online rehabilitation programs for SD in PCa patients. For instance, Schover et al. compared internet-based sexual counseling with traditional face-to-face counseling for couples post-PCa treatment, finding similar improvements in sexual function, satisfaction, and relationship quality.^38^ Patients also expressed a preference for the online option due to its convenience. Additionally, Kang et al. found that online interventions for cancer patients’ sexual health effectively addressed their concerns.^39^ Together, these studies, along with our findings, support the viability of virtual programs as an effective, accessible method of delivering sexual healthcare for PCa patients.

The COVID-19 pandemic accelerated the adoption of telehealth, which had previously faced slow uptake due to factors like staff reluctance, funding challenges, and poor implementation strategies.^40^^,^^41^ However, the pandemic created unique circumstances that pushed healthcare organizations to integrate virtual care, demonstrating that telehealth can offer convenience, flexible access, and reduced travel costs without negatively affecting patient outcomes.^42^^,^^43^ Additionally, virtual care can also help reduce stigma associated with in-person sexual health programs, offering patients confidential, effective support from home.

A distinguishing feature of SHAReClinic is its use of asynchronous care, which differs from traditional telehealth models that rely on real-time synchronous interactions. By offering flexible, self-guided modules and asynchronous messaging with coaches, SHAReClinic reduces resource demands on healthcare providers while maintaining patient engagement and satisfaction. This care model may offer important economic and scalability advantages for institutions looking to expand sexual health services without a corresponding increase in staffing or infrastructure requirements.

### Limitations and future directions

This study’s retrospective design limits the ability to establish causality, and the reliance on self-reported measures of sensitive topics like sexual health may introduce response bias. Participants may underreport or overreport their symptoms due to social desirability or recall bias. Additionally, as a retrospective review of routine clinical care, our sample was restricted to patients seen during a specific time window, and analyses were limited to data captured in existing records. This may have constrained the scope of variables available for comparison and reduced control over confounding factors.

An important limitation is that the imbalance in treatment modality between groups is a limitation. Radiation patients were only represented in the SHAReClinic cohort (n = 8), while all PCRC patients underwent surgery. Due to the small number of radiation patients, treatment type could not be included in the multivariable analysis. This imbalance may bias sexual dysfunction outcomes, particularly in the early post-treatment period when recovery trajectories differ between surgery and radiation. Future studies with larger, treatment-balanced cohorts will be needed to fully evaluate the impact of treatment modality.

This study was not designed to evaluate patient-level predictors of success, which should be investigated in larger, prospective cohorts. In addition, the lack of diversity in the study sample, which was predominantly Caucasian, reflects the demographics of the cancer center, reducing the generalizability of the findings to more diverse populations. The setting at a large, urban teaching hospital may limit its applicability to rural or less-resourced areas, particularly for the in-person PCRC program. Furthermore, the non-random assignment of patients to PCRC or SHAReClinic introduces potential selection bias, though this real-world setup minimizes biases related to active recruitment or research team involvement. Additionally, since this study utilized retrospective chart reviews from patients already attending the PCRC or SHAReClinic, it lacks data on patients who opted out of participation altogether. This prevents assessment of potential differences between participants and non-participants, limiting our understanding of broader patient needs, preferences, or barriers to joining sexual health clinics.

## Conclusion

This study aimed to assess whether outcomes in the virtual SHAReClinic were comparable to those observed in the in-person PCRC in improving sexual health outcomes for PCa survivors. The results suggest that SHAReClinic is likely equally effective in managing SD, reducing distress, and improving overall quality of life. These findings contribute to the growing body of evidence supporting the use of online intervention in oncology care, providing valuable insights for healthcare systems aiming to improve patient outcomes while managing financial and logistical constraints.

## Data Availability

The data presented in this study are available on request from the corresponding author.
